# Radioactive contamination in the Tokyo metropolitan area in the early stage of the Fukushima Daiichi Nuclear Power Plant (FDNPP) accident and its fluctuation over five years

**DOI:** 10.1371/journal.pone.0187687

**Published:** 2017-11-14

**Authors:** Masanobu Ishida, Hideo Yamazaki

**Affiliations:** Graduate School of Science and Engineering, Kindai University, Higashiosaka, Osaka, Japan; University of South Carolina, UNITED STATES

## Abstract

Radioactive contamination in the Tokyo metropolitan area in the immediate aftermath of the Fukushima Daiichi Nuclear Power Plant (FDNPP) accident was analyzed via surface soil sampled during a two-month period after the accident. ^131^I, ^134^Cs, and ^137^Cs were detected in these soil samples. The activity and inventory of radioactive material in the eastern part of Tokyo tended to be high. The ^134^Cs/^137^Cs activity ratio in soil was 0.978 ± 0.053. The ^131^I/^137^Cs ratio fluctuated widely, and was 19.7 ± 9.0 (weighted average 18.71 ± 0.13, n = 14) in the Tokyo metropolitan area. The radioactive plume with high ^131^I activity spread into the Tokyo metropolitan area and was higher than the weighted average of 6.07 ± 0.04 (n = 26) in other areas. The radiocesium activity and inventory surveyed in soil from a garden in Chiyoda Ward in the center of Tokyo, fell approximately 85% in the four months after the accident, and subsequently tended to rise slightly while fluctuating widely. It is possible that migration and redistribution of radiocesium occurred. The behavior of radiocesium in Tokyo was analyzed via monitoring of radiocesium in sludge incineration ash. The radiocesium activity in the incineration ash was high at wastewater treatment centers that had catchment areas in eastern Tokyo and low at those with catchment areas in western Tokyo. Similar to the case of the garden soil, even in incineration ash, the radiocesium activity dropped rapidly immediately after the accident. The radiocesium activity in the incineration ash fell steadily from the tenth month after the accident until December 2016, and its half-life was about 500 days. According to frequency analysis, in central Tokyo, the cycles of fluctuation of radiocesium activity in incineration ash and rainfall conformed, clearly showing that radiocesium deposited in urban areas was resuspended and transported by rainfall run-off.

## Introduction

The tsunami triggered by the East Japan Great Earthquake Disaster of March 11, 2011 cut off all electric power to Fukushima Daiichi Nuclear Power Plant (FDNPP). No. 1, No. 2, and No. 3 reactors lost their cooling capability, their nuclear fuel went into melt down and their reactor vessels were destroyed between March 12 and 15 [1–6]. As a result, part of the radioactive material discharged into the atmosphere was deposited across eastern Japan [7–11]. Based on monitoring data from each location, it is assumed that radioactive material in the plume that spread into the Tokyo metropolitan area on March 16 and 22 was deposited on the ground via rainfall. The timing and route of the spread of the radioactive plume have been variously hypothesized, but have not been clarified in detail as of yet [12–17]. When we started our measurements, almost no public information about the radioactive contamination in the Tokyo metropolitan area and Kanto district had been shared.

The world has experienced nuclear power plant accidents before, including nuclear fuel melt down at Three Mile Island (1979) [18] and Chernobyl (1986) [19,20]. Six years after the FDNPP accident in 2011, much about the behavior of radioactive material in the environment has been clarified [1,21]. The FDNPP accident caused the nuclear fuel meltdown simultaneously in three reactors, discharging a large quantity of radioactive material into the environment [6–11]. One characteristic of the FDNPP accident is that a large quantity of radioactive material was discharged into the topographically and meteorologically complex natural environment of Japan. Because many of the urban areas are formed in the plains at the foot of steep mountainous areas, they are frequently affected by heavy rains such as typhoons. Another major characteristic is that the plume containing a large amount of radioactive material drifted into the Tokyo metropolitan area [16,21–24]. The capital, Tokyo, where over 30 million residents live, is located south of Kanto Plain, about 230 km southwest of FDNPP. Conditions and radioactive contamination shortly after the FDNPP accident have been reported in a variety of publications [2–5]. However, these include almost no discussion of the fact that radioactive materials were carried into the Tokyo metropolitan area, which is a densely populated urban area and the socio-economic and cultural core of Japan, nor of any evaluation of the fluctuations in the radioactive material in Tokyo’s urban districts.

This study surveyed the distribution of radioactive materials in soil to evaluate the fluctuation of radioactive contamination in the Tokyo metropolitan area and the Kanto district in the immediate aftermath of FDNPP accident. Changes in radionuclide activities and inventories in soil between the time of the accident and the present time in the central part of Tokyo were monitored to clarify the behavior of the radioactive materials in the urban distritcts. Moreover, the behavior of radionuclides in the urban environment, which is covered by concrete and asphalt, was analyzed based on changes in the radiocesium activity in incineration sludge ash discharged from Tokyo’s wastewater treatment centers [25].

## Sampling and methods

### Soil sampling

Most soil samples were taken from the private site. These collections were approved by the owner of the land. In the case of collection at public places, sampling was carried out with the permission of the administrator when necessary.

**Fig 1:**
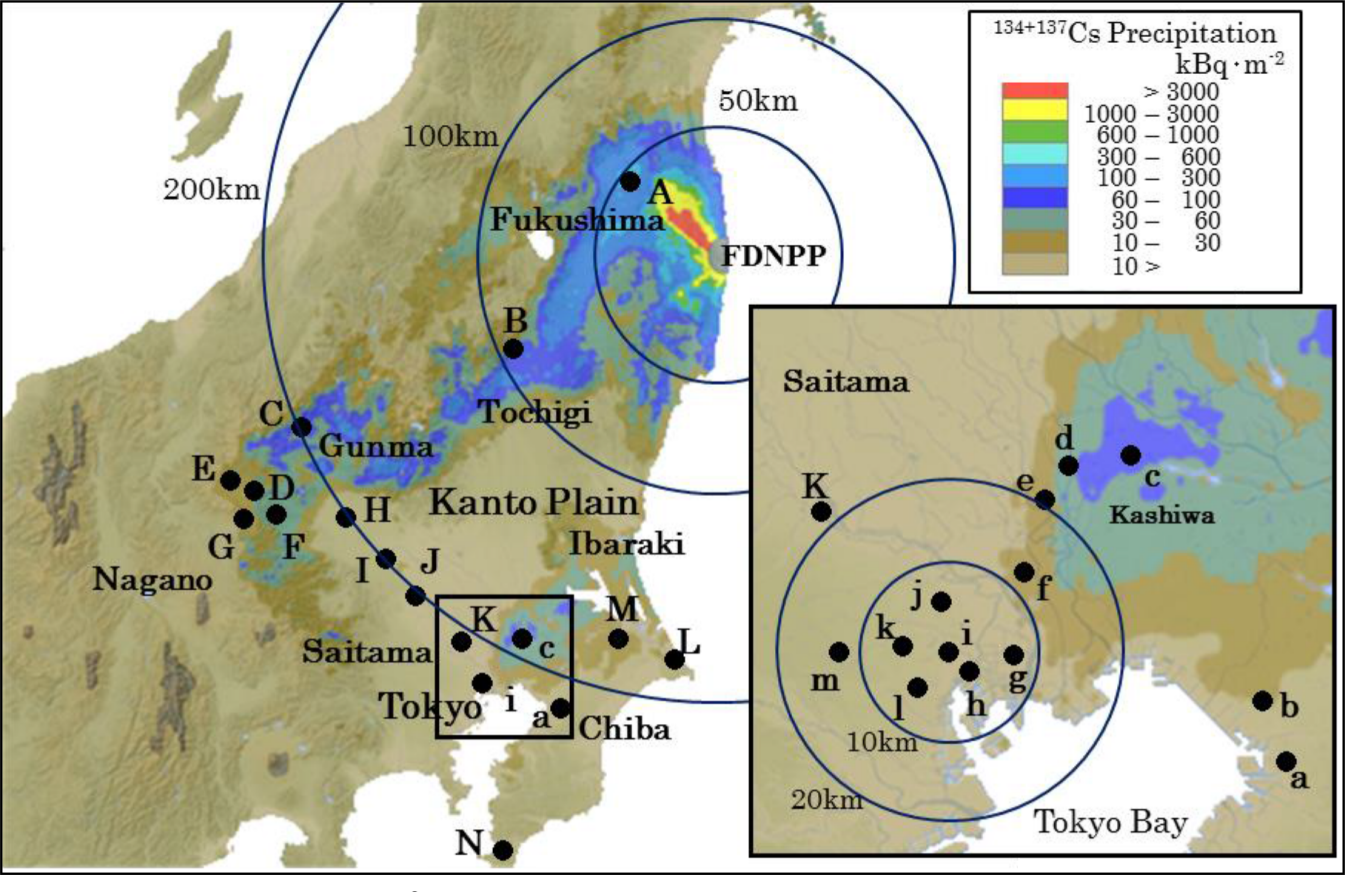
Sampling sites of soil samples. Geographical distribution of the ^134+137^Cs precipitation referred to the aircraft monitoring results by the MEXT of Japan on December 16, 2011 [30]. Adapted from ‘Extension Site of Distribution Map of Radiation Dose, etc.’ (http://ramap.jmc.or.jp/map/).

For this study, soil samples were collected in the Tokyo metropolitan area and the Kanto district during the two months following the FDNPP accident. In general, the samples were obtained from the ground surface along roads where they were not subject to physical turbation. The sampling sites were located at the ground surface of flat gardens by the roadside, and in many cases were hardly affected by the flow of rainwater and were not covered with plants. Soil in Site ‘i’ was sampled in an area 0.5 × 1 m, and the core samples were obtained to a depth of 20 cm, varying the position slightly for each sampling in the area. After sampling, the holes were refilled. There was grass, vegetation, etc. at the sampling locations, and the soil was exposed at the ground surface. The ground was slightly sloped, so it is assumed that when rain falls, rainwater flows gently over the ground surface. Pedestrians could walk on the sampling locations. Surface soil up to 1 cm deep below the surface was measured using a measure and removed from a 10 × 10 cm area. A core sample was collected to a depth of 20 cm using a core sampler with an inner diameter of 5 cm, and sliced to an appropriate thickness to prepare an analytical sample. The soil samples were air-dried and impurities, such as vegetation detritus and rocks with diameters of 1 mm or larger, were removed before their radioactivity was measured. The sampling sites were selected in the Tokyo metropolitan area and Kanto district approximately 200 km from FDNPP, and are shown in Fig 1. Sampling of soil should normally be done by the established IAEA method [26]. However, this method could not be applied to urban regions contaminated with activity, because buildings and roads are interspersed there. The inventory of radionuclides was calculated assuming soil density of 1,300 kg·m^-3^ in compliance with the method of the MEXT of Japan [27], because accurate evaluation of soil density is difficult and many already reported inventories of radiocesium are estimated using this value.

### Measuring radioactivity

The activity concentrations of radionuclides in the soil were quantified by connecting a 4096 multichannel pulse height analyzer (Labo Equipment, MCA600) to a low energy HPGe detector (ORTEC LO-AX/30P) sheathed in 10 cm thick lead. The specimen was sealed inside a plastic container with a diameter of 5.5 cm and depth of 2.0 cm, in preparation measurement by γ-ray spectrometry. The Ge detector calculated the geometric efficiency relative to the sample volume using the American NIST Environmental Radioactivity Standards, SRM 4350B (River Sediment) and SRM 4354 (Freshwater Lake Sediment), and the numerical efficiency was corrected within the range of 2 to 30 g of sample weight [28]. The measurement time was set so that the counting error would be less than 5%, depending on the radioactive intensity of the samples. In this study, ^131^I (364 keV), ^134^Cs (605 keV), and ^137^Cs (662 keV) were quantified. A ^134^Cs solvent with known density was used to correct the sum peak effect for ^134^Cs.

The radioactivity was calculated based on a value for the day the samples were obtained. Late at night on March 15, 2011, the No. 2 reactor went into melt down, causing the discharge of the largest quantity of radionuclides during the FDNPP accident [2,3,6,23], and the radioactivity measured in this study was evaluated based on a value obtained by decay correction at noon on March 16, 2011. Because its half-life is short (8.04 days), ^131^I was not detected from samples collected after late June 2011. The samples that were not analyzed immediately after sampling showed lower measurement precision for ^131^I than for ^134^Cs or ^137^Cs.

### Radioactivity of sludge incineration ash

The radioactivity in incineration sludge ash discharged by Tokyo’s wastewater treatment centers was monitored by Tokyo Metropolitan Government beginning in May 2011, and the results have been provided on the Metropolitan Tokyo website [25]. In most areas of Tokyo, domestic wastewater is mixed with rainwater in the wastewater treatment system and this mixture is fed to the wastewater treatment centers for purification. Radiocesium is very efficiently captured in the sludge that is recovered, which is incinerated and disposed of as incineration ash. Therefore, it can be assumed that the radioactivity of incineration ash discharged from Tokyo’s wastewater treatment centers reflects the quantity of radioactive material deposited in their respective catchment areas [29]. Thus, changes in the radiocesium activity of the sludge incineration ash from the wastewater treatment centers was analyzed to evaluate the distribution and behavior of radiocesium in Tokyo for a five year period after the FDNPP accident.

### Frequency analysis between radioactivity in sludge incineration ash and monthly rainfall

The relationship between the radiocesium activity in the sludge incineration ash and the monthly rainfall in the Tokyo metropolitan area was frequency analyzed using the MATLAB software.

## Results and discussion

### Distribution of radionuclides in soil of the Tokyo metropolitan area in the immediate aftermath of the accident

In this study, surface soil was sampled from inside Tokyo and in the prefectures of Fukushima, Tochigi, Gunma, Ibaraki, Chiba, and Saitama, as shown in Fig 1, between late March, immediately after the accident, and early June. The measurement results are shown in Table 1, as the activity and inventory normalized for the time each was sampled and for March 16, 2011.

**Table 1:** Analytical results of the activity of radionuclides in the soil samples collected in the Tokyo metropolitan area and the Kanto district area.

Prefecture	Site	Description	Collected Date	Taken cm	Decay Corrected Activity Bq·kg^-1^				Ratio for ^137^Cs		Inventory kBq·m^-2^	
					^131^I	^134^Cs	^137^Cs	^134+137^Cs	^131^I	^134^Cs	^131^I	^134+137^Cs
Fukushima	A	Schoolyard (1)	2011/3/19	0–1	122000	13900	14100	28000	8.652	0.986	1590	363
		Schoolyard (2)	2011/4/27	0–1	142000	21400	21900	43300	6.484	0.977	1850	563
Tochigi	B	Pasture	2011/6/4	0–1	43600	11100	11300	22400	3.858	0.982	566	290
		Garden	2011/6/13	0–1	24500	3020	3150	6170	7.778	0.959	318	80.2
		Farmland	2011/6/13	0–1	91800	6730	6810	13500	13.48	0.988	1190	176
Nagano	G	Plateau Forest	2011/4/28	0–1	513	550	557	1110	0.920	0.986	6.7	14.4
		Hotel Garden	2011/4/28	0–1	3350	726	755	1480	4.439	0.962	43.6	19.3
Gunma	C	Paddy Field	2011/4/30	0–1	5640	1830	1850	3680	3.049	0.989	73.3	47.9
	D	Forest	2011/3/26	0–1	582	67.4	70.7	138	8.232	0.953	7.6	1.8
		Farmland	2011/4/29	0–1	1630	183	184	367	8.854	0.996	21.2	4.8
				1–3	nd	nd	4.5	4.5	-	-	-	0.1
				3–5	nd	nd	nd	nd	-	-	-	-
	E	Farmland	2011/4/29	0–1	4360	1760	1720	3480	2.535	1.023	56.7	45.1
	F	Plateau Forest	2011/5/1	0–1	2050	824	810	1630	2.531	1.018	26.6	46.2
	H	Road Side	2011/4/28	0–1	3860	740	745	1490	5.178	0.993	50.1	19.3
Saitama	I	Rode Side	2011/4/28	0–1	nd	151	154	305	-	0.979	-	4.0
	J	Road Side	2011/4/28	0–1	3130	204	207	412	15.10	0.986	40.7	5.4
	K	River Bank	2011/4/10	0–1	6130	248	252	500	24.29	0.981	79.6	6.5
	e	Garden	2011/5/19	0–1	25200	1060	1080	2140	23.33	0.981	328	27.7
Ibaraki	L	Garden	2011/4/20	0–1	4850	225	237	462	20.45	0.949	63.1	6.0
	M	Garden	2011/4/20	0–1	8420	105	108	213	78.11	0.977	109	2.8
Chiba	N	Garden	2011/4/10	0–1	582	59.7	69.2	129	8.41	0.863	7.6	1.7
	a	Road Side	2011/4/11	0–1	7020	672	672	1340	10.45	1.000	91.3	17.5
	b	Road	2011/4/11	0–1	12100	2460	2510	4970	4.821	0.980	157	64.6
	c	Garden	2011/5/19	0–1	40700	5300	5330	10600	7.636	0.994	529	138
		Road Sludge	2011/6/11	0–1	114000	33900	33900	67800	3.363	1.000	1480	881
		Road Side	2011/6/11	0–1	28500	17700	17700	35400	1.610	1.000	371	460
	d	Road Side	2011/5/19	0–1	26100	3210	3190	6400	8.182	1.006	340	83.2
Tokyo	f	Road Side	2011/5/19	0–1	56900	3470	3410	6880	16.69	1.018	740	89.4
	g	Garden (1)	2011/4/16	0–1	25700	1630	1610	3240	15.96	1.012	334	42.1
			2011/4/27	0–1	77500	3560	3600	7160	21.53	0.989	1010	93.1
		Garden (2)	2011/5/25	0–1	27700	1710	1740	3450	15.92	0.983	360	44.8
	h	Road Side	2011/4/10	0–1	10100	588	580	1170	17.41	1.014	131	15.2
	i	Garden	2011/4/10	0–1	18500	966	962	1930	19.23	1.004	240	25.1
			2011/4/28	0–1	53700	1180	1180	2360	45.51	1.000	698	30.6
				1–3	1360	43.6	62.5	106	21.76	0.698	35.0	3.0
				3–5	nd	nd	1.4	1.4	-	-	-	0.02
	j	Garden	2011/4/10	0–1	20000	644	683	1330	29.29	0.942	259	17.2
	k	Road side (1)	2011/6/2	0–1	18200	1780	1760	3540	10.34	1.011	237	46.1
		Road Side (2)	2011/6/2	0–1	7780	475	473	948	16.44	1.004	101	12.3
		Road Side (3)	2011/6/2	0–1	11600	514	536	1050	21.66	0.959	151	13.6
	l	Road Side	2011/6/2	0–1	11100	1230	1220	2450	9.098	1.008	145	31.9
	m	Garden	2011/5/19	0–1	6740	432	442	874	15.24	0.976	87.6	11.4

Decay corrected activity as corrected on the March 16, 2011. Inventory was caluculated assuming 1,300 kg·m^-3^ for the soil density. At Site 'i', measurements continued from April 10, 2011 until August 16, 2016. The results are shown in Fig 3. nd: Not Detected (^131^I; < 2 Bq·kg^-1^, ^134^Cs and ^137^Cs; < 0.6 Bq·kg^-1^).

Regarding the radioactive contamination of soil caused by the FDNPP accident, the geographical heterogeneity of the radioactive materials deposited on the ground surface was uneven and narrow ranging. This based on the analytical results of five core samples taken from the four corners and center of a 10 × 10 m uncultivated paddy field at Site ‘B’ in Tochigi, shown in Fig 1. More than 95% of radiocesium deposited on the soil surface at these points was retained in the upper 5 cm depth from the surface of the 20 cm deep core, while the standard deviation of the inventory was ± 28.9% (125–280 kBq·m^-2^, n = 5). The uneven distribution of radioactive materials deposited on the surface in this way was confirmed at many points. This suggests the possibility that radioactive materuials in the plume were not uniformly mixed physically while they were transported in the atmosphere.

### Geographical distribution of radionuclides

The geographical distribution of radioactive materials in the soil in the Tokyo metropolitan area and Kanto district, as shown in Fig 1, was interpolated from the results of aerial monitoring by the MEXT that were announced at December 16, 2011 [30]. The radionuclide inventory in the Kanto district was high in Tochigi, northwest Gunma, eastern Nagano, and northern Chiba, but lower in Saitama and southern Chiba. In the Tokyo metropolitan area, it was high in part of eastern Tokyo. The contamination level was extremely high in a roadside ditch sludge in Kashiwa City (Site ‘c’) of northern Chiba, because radionuclides were concentrated in the drainage. Excluding this area, places showing the highest inventories in the Tokyo metropolitan area were 1010 kBq·m^-2^ in Koto Ward (Site ‘g’) for ^131^I and 460 kBq·m^-2^ in Kashiwa City (Site ‘c’) for ^134+137^Cs (total of ^134^Cs and ^137^Cs). In central Tokyo (Sites ‘f’ to ‘m’, n = 12), for ^131^I, it was 695 ± 291 kBq·m^-2^, and for ^134+137^Cs, it was 74.9 ± 27.3 kBq·m^-2^, which are about 40% and 20% of the ^131^I and ^134+137^Cs levels in Fukushima City (Site ‘A’), respectively. This result also suggests that the plume, which includes radioiodine activities much higher than those of radiocesium during the immediate aftermath of the accident, spread into the Tokyo metropolitan area [16,22–24].

### ^131^I /^137^Cs and ^134^Cs /^137^Cs activity ratios

**Fig 2:**
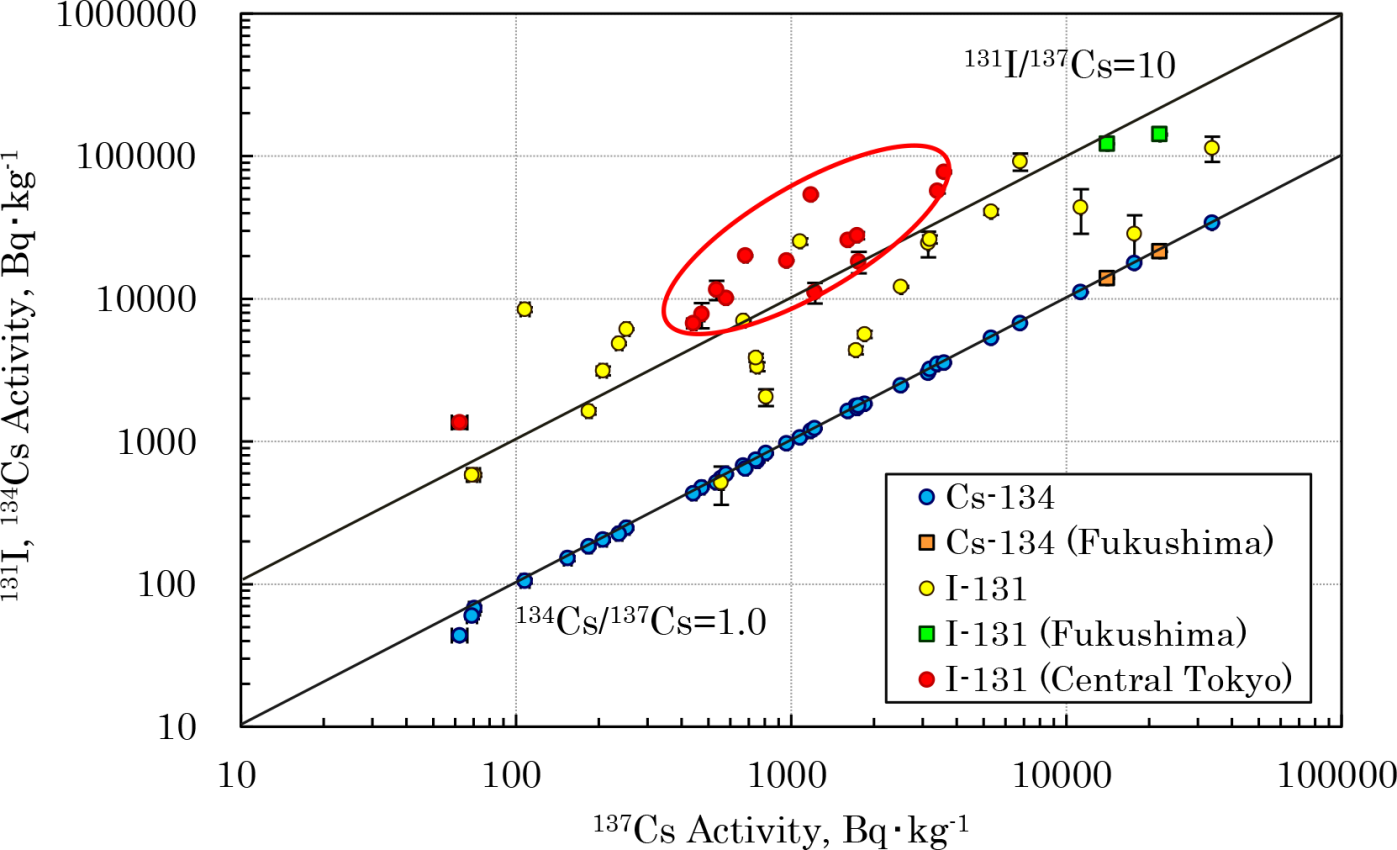
Activity ratios of ^131^I/^137^Cs and ^134^Cs/^137^Cs in the soil collected from eastern Japan.

The isotope ratios are discussed in relation to the source of the radionuclides [23,31–33]. As shown in Table 1 and Fig 2, the ^134^Cs/^137^Cs activity ratio was determined to be 0.989 ± 0.017 (weighted average 0.992 ± 0.002, n = 40). This value conformed closely to the ^134^Cs/^137^Cs ratio 1.0 of radiocesium discharged due to the FDNPP accident within the limits of counting errors [32,33]. The ^131^I/^137^Cs activity ratio caused by the accident has been reported to be about 10 [6,7], but in Fukushima and Tochigi, which are near the FDNPP, it was 8.05 ± 3.53 (weighted average 7.64 ± 0.07, n = 5), indicating an activity ratio that approaches this. But in Nagano, Gunma and southern Chiba (Site ‘N’) which are far from the FDNPP, it was 4.91 ± 2.95 (weighted average 3.48 ± 0.09, n = 9), and in the Saitama, northern Chiba, Ibaraki and Tokyo metropolitan area, it was 18.94 ± 15.38 (weighted average 9.67 ± 0.07, n = 25), indicating significant regional differences. As shown in Fig 2, the ^131^I/^137^Cs ratio was high especially in the central part of Tokyo. It is highly likely that such differences in ^131^I/^137^Cs ratios reflect differences in the physical and chemical behavior of iodine and cesium in the transport and deposition processes of the radioactive plume. From the vertical distribution of radioiodine and radiocesium at Sites ‘D’ and ‘i’ (Table 1), there is a small possibility that differences in their behavior in the soil after deposition on the ground surface had an effect on the measured ^131^I/^137^Cs ratios. In other words, it is assumed that the ^131^I/^137^Cs ratio was extremely high in the Tokyo metropolitan area, because a plume retaining a relatively high ^131^I activity spread into Tokyo. This indicates that the radiocesium deposited preferentially compared to ^131^I in the process of a plume moving from FDNPP [16,22–24].

### Change over time of the radionuclide activity and inventory in garden soil in central Tokyo

**Fig 3:**
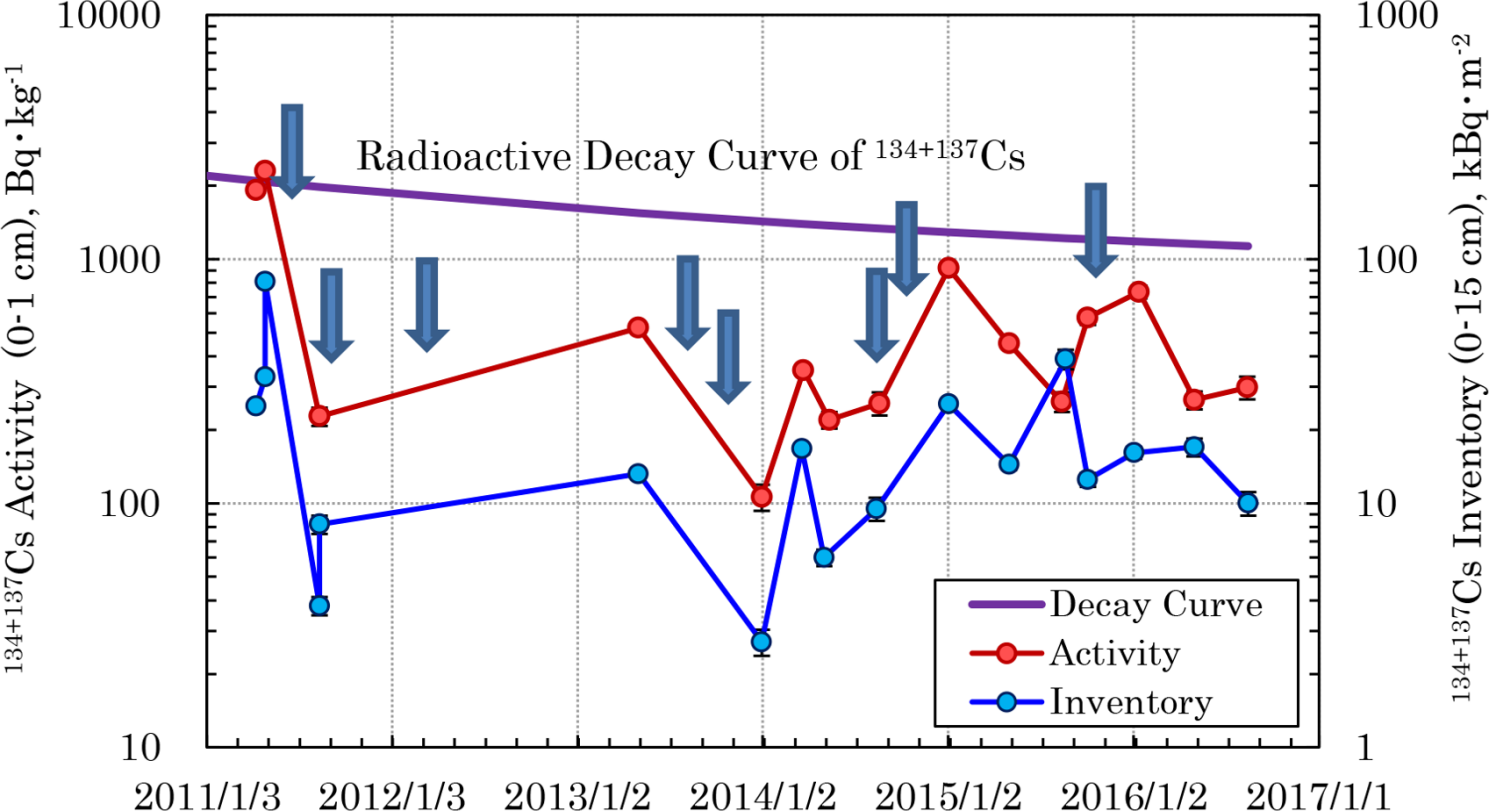
Temporal changes in the radioactivity and inventory of ^134+137^Cs in the surface soil. The radioactivity and inventory were measured for 0–1 cm and 0–15 cm layers at Site ‘i’, respectively. The radioactive decay curve shown assumes equal radioactivity of ^134^Cs and ^137^Cs immediately after the accident. Vertical arrows show heavy rain of more than 75 mm in the Tokyo metropolitan area.

At Site ‘i’ in a garden in Chiyoda Ward, located in the center of Tokyo, the radioactivity and inventory of radionuclides in the soil were surveyed continuously. The first sampling was done on April 10, 2011, and the ^131^I (2,140 Bq·kg^-1^) and ^134+137^Cs (1,900 Bq·kg^-1^) were detected at high activities. Both nuclides were concentrated mostly on the surface, as other locations [34, 35]. Fig 3 shows the temporal changes in radioactivity in the layer 1 cm from the surface and of inventory in all layers (depth from 0–15 cm) of ^134+137^Cs from the accident. The decay curve of radiocesium at the time when it was hypothesized that the activities of ^134^Cs and ^137^Cs deposited in the immediate aftermath of the accident were the same is also shown in Fig 3. From April 10 to 28, 2011, in the immediate aftermath of the accident, the activities and inventories of ^131^I and ^134+137^Cs increased abruptly (Table 1). If this increase is not a result of uneven distribution of radionuclides in the samples, the spread of the radionuclides into the Tokyo metropolitan area from the FDNPP probably continued at this time [24]. However, the ^134+137^Cs activity fell to about 10% of its initial value by August 13, while ^131^I was not detected at the time, so we assumed that the spread of new radionuclides from the nuclear reactor had stopped.

The adsorption behavior of radionuclides to clay minerals has been studied for many years and it is well known that radiocesium penetrates layers of clay minerals where it undergoes strong ionic adsorption and is bound by the soil particles [36–45]. The torrential rainfall from Typhoon 2 on May 29 and 30, 2011 washed out soil particles that had adsorbed radiocesium, such that it is undeniable that this may have reduced the radiocesium activity. However, because heavy rainfall has fallen in the Tokyo metropolitan area since that time, soil run-off due to rainfall alone cannot account for the rapid decline in the radioactivity in the immediate aftermath of the accident. This fact suggests that radiocesium that was deposited immediately after the FDNPP accident could have contained a large quantity of a highly mobile form radiocesium. In other words, it can be assumed that this radiocesium existed as a chemical species that is not easily held in the soil. The radiocesium activity and inventory in the garden soil of Site ‘i’ appear to have fluctuated widely, as they steadily increased over time beginning in the second year after the accident. The radioactivity level predicted based on radioactive decay beginning in the third year after the accident is shown in Fig 3, and indicates that it is possible that the movement and recycling of radiocesium at this location were balanced. The changes over time in radioactivity of the soil in the Tokyo metropolitan area could possibly reflect the effects of uneven distribution due to the sampling locations, so fluctuation in radioactivity over time was studied using sludge incineration ash.

### Behavior of radionuclides in the Tokyo metropolitan area shown by sludge incineration ash from wastewater treatment centers

**Fig 4:**
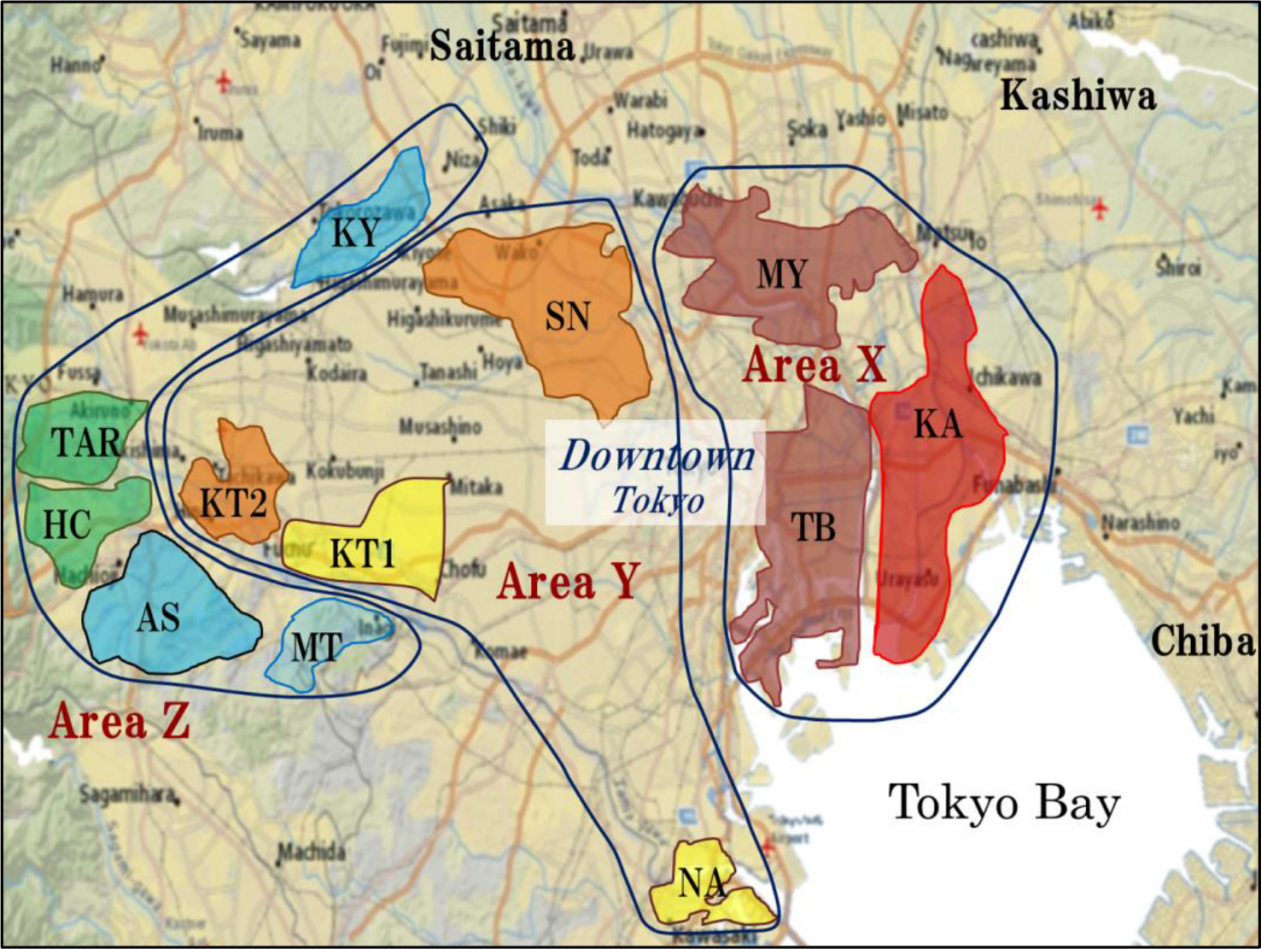
Sites of Tokyo’s wastewater treatment centers and their catchment areas. Base map is from website USGS National Map Viewer. (http://viewer.nationalmap.gov/viewer/).

Regarding the process of movement of radioactive materials that have pervaded the environment from their catchment areas via rainfall and rivers, various measurement data and the results of simulations based on these have been reported [46–53]. A detailed comparison of model predictions and actual observed results in Sweden after the Chernobyl accident was also conducted on the ^137^Cs migration process by sewage treatment system in the urban environment [54]. However, the fluctuation of radionuclides in urban areas remains almost completely unknown. Radiocesium in sludge incineration ash discharged from wastewater treatment centers in Tokyo was used as a tracer in this study to evaluate the fluctuation of radiocesium in the urban environment. Wastewater treatment centers at 12 locations in Tokyo, shown in Fig 4, have published changes over time in the radiocesium activities in combined sludge incineration ash from May 2011 up to the present time [25]. Of the wastewater treatment centers listed on the website, the Nanbu wastewater treatment center (NA) also processes sludge it receives from other treatment plants [29].

### Geographical distribution and change over time of radiocesium activity in combined sludge incineration ash

**Fig 5:**
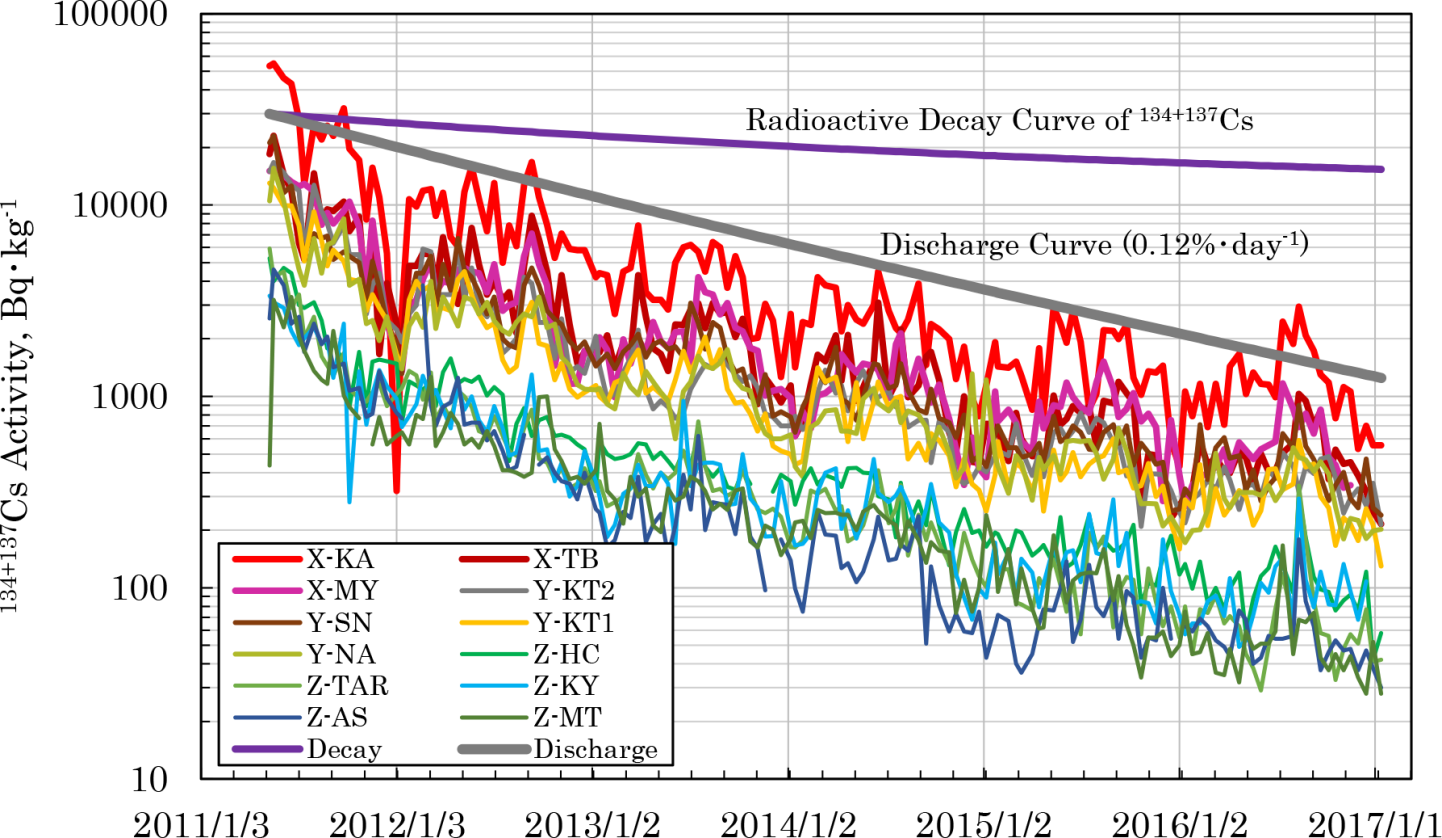
Temporal changes of the ^134+137^Cs radioactivity in the incineration ash from Tokyo’s wastewater treatment centers.

Fig 5 shows the changes over time in radiocesium activity in sludge incineration ash discharged from each wastewater treatment center. In May 2011, when the measurement values were published, the Kasai center (KA), whose catchment area is in eastern Tokyo, showed the highest activity, at about 70,000 Bq·kg^-1^, whereas the Miniami Tama center (MT) in the southwest showed the lowest activity of about 3,000 Bq·kg^-1^. The radiocesium activity in the sludge incineration ash decreased from the east to the west. This finding conforms to aerial monitoring and soil measurement values, showing that the radiocesium activity in sludge incineration ash reflects the radioactivity level of the catchment area of each wastewater treatment center. The radiocesium activity in sludge incineration ash decayed almost exponentially with the passage of time. Its decay velocity was higher than the radioactive decay of radiocesium discharged by the FDNPP accident, showing that radiocesium in the environment was effectively removed by the wastewater treatment systems. The half-life of the eliminated radiocesium was estimated at between 460 and 580 days. Not all the radiocesium deposited in the Tokyo metropolitan area was discharged through the wastewater treatment systems, but in the center of Tokyo, from 0.12–0.15% of radiocesium deposited on the surface was removed each day.

Table 2 shows the annual average activity (arithmetic mean value) of radiocesium in sludge incineration ash discharged from the wastewater treatment centers. Furthermore, Table 2 also shows the annual discharge quantity of incineration sludge ash and the annual discharge quantity of radiocesium calculated based on the quantity and radioactivity discharged. The radiocesium activity and quantity discharged varied greatly according to the catchment area of the wastewater treatment centers. The quantity of radiocesium discharged as sludge incineration ash from immediately after the accident until December 2015 reached 0.60 TBq (× 10^12^ Bq: not decay corrected). This value includes the value of the NA plant that also accepted sludge from other plants in central Tokyo. The radioactivity level in the Tokyo metropolitan area was categorized into regions X, Y, and Z, shown in Fig 4, based on the levels of radiocesium activity in the sludge incineration ash.

**Table 2:** Annual changes in the activity and amount of radiocesium contained in the sludge incineration ash discharged from the wastewater treatment plants.

Wastewater Treatment Plants			Activity of ^134+137^Cs in Ash Bq·kg^-1^ (Annual Average)					Annual Discharged of Ash ton·yr^-1^						Annual Discharged Amount of ^134+137^Cs by Ash TBq (×10^12^ Bq)					
Area	Code	Name	2011	2012	2013	2014	2015	2011	2012	2013	2014	2015	Total	2011	2012	2013	2014	2015	Total
X	KA	Kasai	26159	8770	4202	2490	1606	5260	6597	5720	6444	6159	30180	0.1376	0.0579	0.0240	0.0160	0.0099	0.2454
	TB	Tobu	9649	4146	2082	1271	690	6814	8515	8003	8535	8259	40126	0.0658	0.0353	0.0167	0.0108	0.0057	0.1343
Y	MY	Miyagi	9776	3488	2060	1099	842	1320	1543	1095	1466	1319	6743	0.0129	0.0054	0.0023	0.0016	0.0011	0.0233
	KT2	Kitatama 2	8551	2997	1217	818	528	319	302	375	368	339	1703	0.0027	0.0009	0.0005	0.0003	0.0002	0.0046
	SN	Shingashi	8108	3262	1671	988	559	2850	3539	3666	3668	3603	17326	0.0231	0.0115	0.0061	0.0036	0.0020	0.0464
	KT1	Kitatama 1	6419	2413	1166	737	400	982	1332	1323	1321	1328	6286	0.0063	0.0032	0.0015	0.0010	0.0005	0.0126
	NA	Nanbu	5965	2410	1098	716	461	10218	13295	12685	13201	12990	62389	0.0609	0.0320	0.0139	0.0094	0.0060	0.1223
Z	HC	Hachioji	2538	956	414	302	164	446	516	287	466	401	2115	0.0011	0.0005	0.0001	0.0001	0.0001	0.0019
	TAR	Tamagawa	2217	759	358	221	107	892	1183	1288	1220	1236	5818	0.0020	0.0009	0.0005	0.0003	0.0001	0.0037
	KY	Kiyose	1829	751	351	242	135	1268	1608	1606	1635	1607	7722	0.0023	0.0012	0.0006	0.0004	0.0002	0.0047
	AS	Asagawa	1940	673	245	131	72	213	302	489	358	396	1758	0.0004	0.0002	0.0001	0.0000	0.0000	0.0008
	MT	Minamitama	633	607	296	192	102	519	676	639	669	657	3160	0.0003	0.0004	0.0002	0.0001	0.0001	0.0011
							Total	31099	39409	37176	39350	38292	185327	0.3155	0.1495	0.0665	0.0438	0.0259	0.6012

These data were estimated by the published values in website od the Tokyo's wastewater treatment centers. The values for 2011 were obtained by integrating from April to December.

### Estimating radiocesium balance in the Tokyo metropolitan area by the sludge incineration ash wastewater treatment centers

The percentage of radiocesium deposited in the immediate aftermath of the FDNPP accident that was discharged through wastewater treatment centers was estimated. Table 3 shows the total quantity of radiocesium discharged by each wastewater treatment center up to the end of December 2015, decay corrected to March 16, 2011 in the immediate aftermath of the accident. The quantity of ^134+137^Cs deposited in the region studied was estimated from the results of aerial monitoring in October 2011 as 2.46 TBq [30], and from values measured in the soil, it was estimated as 5.35 TBq, despite the inadequate number of samples. The quantity discharged by sludge incineration ash was 0.55 TBq, so the percentage of radiocesium that was washed-out as run-off by wastewater treatment systems until the end of 2015 was calculated to be approximately 22% from aerial monitoring and approximately 10% from soil measurements. Either this means that more than approximately 80% of the radiocesium deposited at the time of the accident is still held in the soil. The remaining approximately 20% has been washed-out of the soil as run-off through a route other than wastewater treatment centers.

**Table 3:** Estimating the radiocesium balance in the Tokyo metropolitan area from the wastewater treatment systems.

Wastewater Treatment Centers			Catchment Area km^2^ (s)	Radiocesium Discharged Amount as Ash	Estimated Inventory from the MEXT Aircraft Monitoring		Estimated Inventory from the Soil Radioactivity		Estimated Outflow of the Past Five Years (%)	
Area	Code	Name		TBq (t)	kBq·m^-2^	TBq (u)	kBq·m^-2^	TBq (v)	(t/u)	(t/v)
X	KA	Kasai	48.93	0.280	25	1.223	50	2.447	26.7	13.3
	TB	Tobu	35.12	0.157	7	0.246	40	1.405	72.3	12.7
	MY	Miyagi	16.87	0.027	6	0.101	20	0.101	30.6	9.2
Y	TK2	Kitatama 2	27.44	0.005	5	0.137	-	-	4.5	-
	SN	Shingashi	28.35	0.054	5	0.142	7	0.199	43.6	31.2
	TK1	Kitatama 1	51.23	0.015	5	0.256	11	0.564	8.5	3.0
	NA	Nanbu	4.37	0.143	5	0.022	-	-	653.1	-
Z	HC	Hachioji	85.33	0.002	1	0.085	-	-	3.0	-
	TAR	Tamagawa	93.49	0.004	1	0.094	-	-	5.3	-
	KY	Kiyose	80.42	0.006	1	0.080	5	0.402	7.8	1.6
	AS	Asagawa	39.02	0.001	1	0.039	-	-	2.8	-
	MT	Minamitama	59.00	0.001	1	0.059	-	-	2.5	-
Total or Average			542	0.552	4.5 (u/s)	2.46	20.5 (v/s)	5.35	22.4	10.1

Radiocesium discharged amount as ash was obtained by decay correcting an annual discharged amount on March16, 2011. Radioactivities of ^134^Cs and ^137^Cs immediately after the accident were assumed to be equal, as shown in Table 1. The values of the Nanbu Plant (NA) were excluded because there is sewage processing from other facilities. Since there is no data that can be referenced, the Nanbu, Hachioji, Tamagawa, Asagawa and Minamitamagawa were excluded from the calculation of the total and average. Precipitations of radiocesium in area Z are estimated values.

### Run-off process of radiocesium via sludge incineration ash

**Fig 6:**
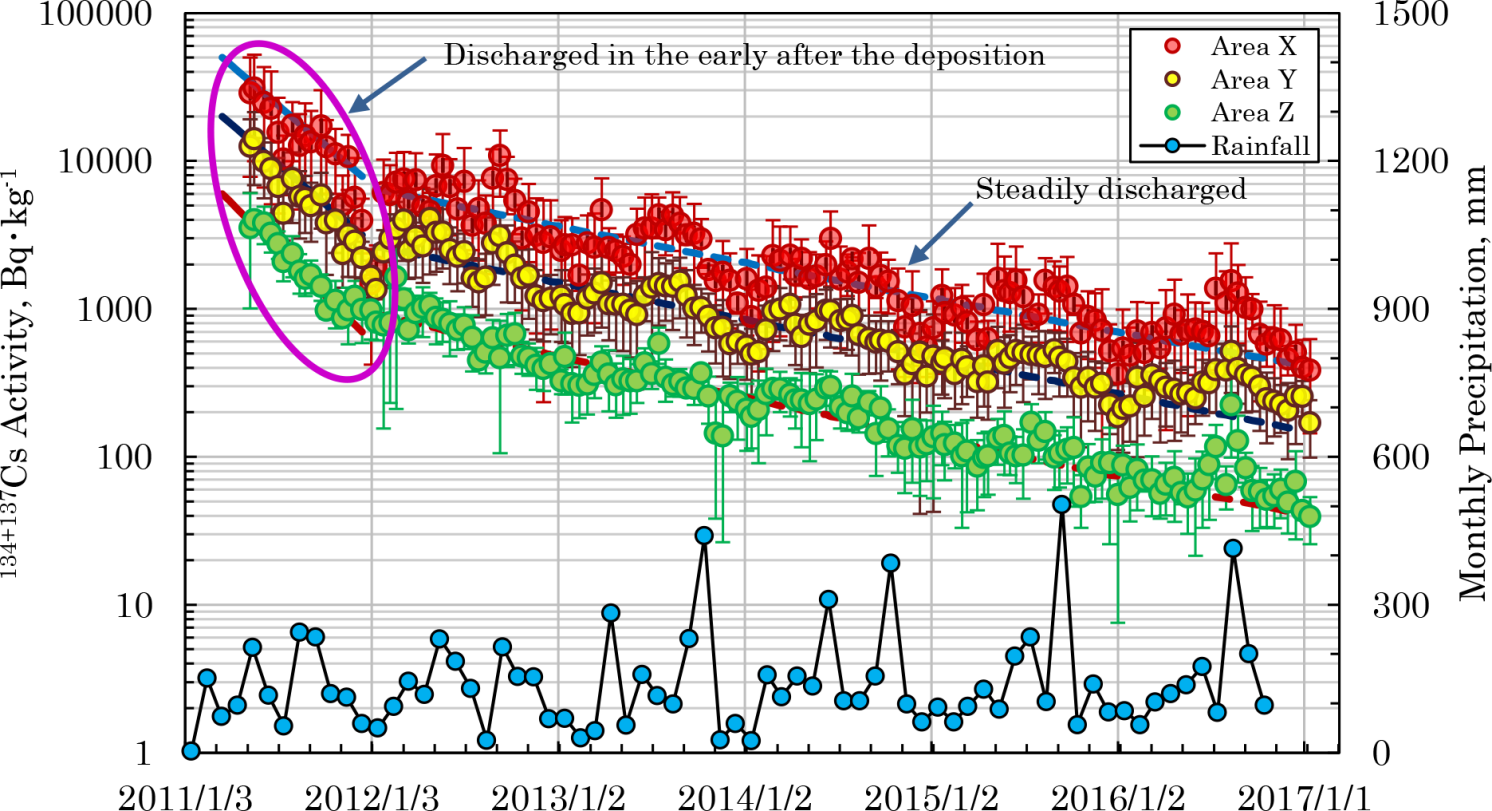
Temporal changes of the ^134+137^Cs radioactivity in the incineration ash and monthly precipitation.

**Fig 7:**
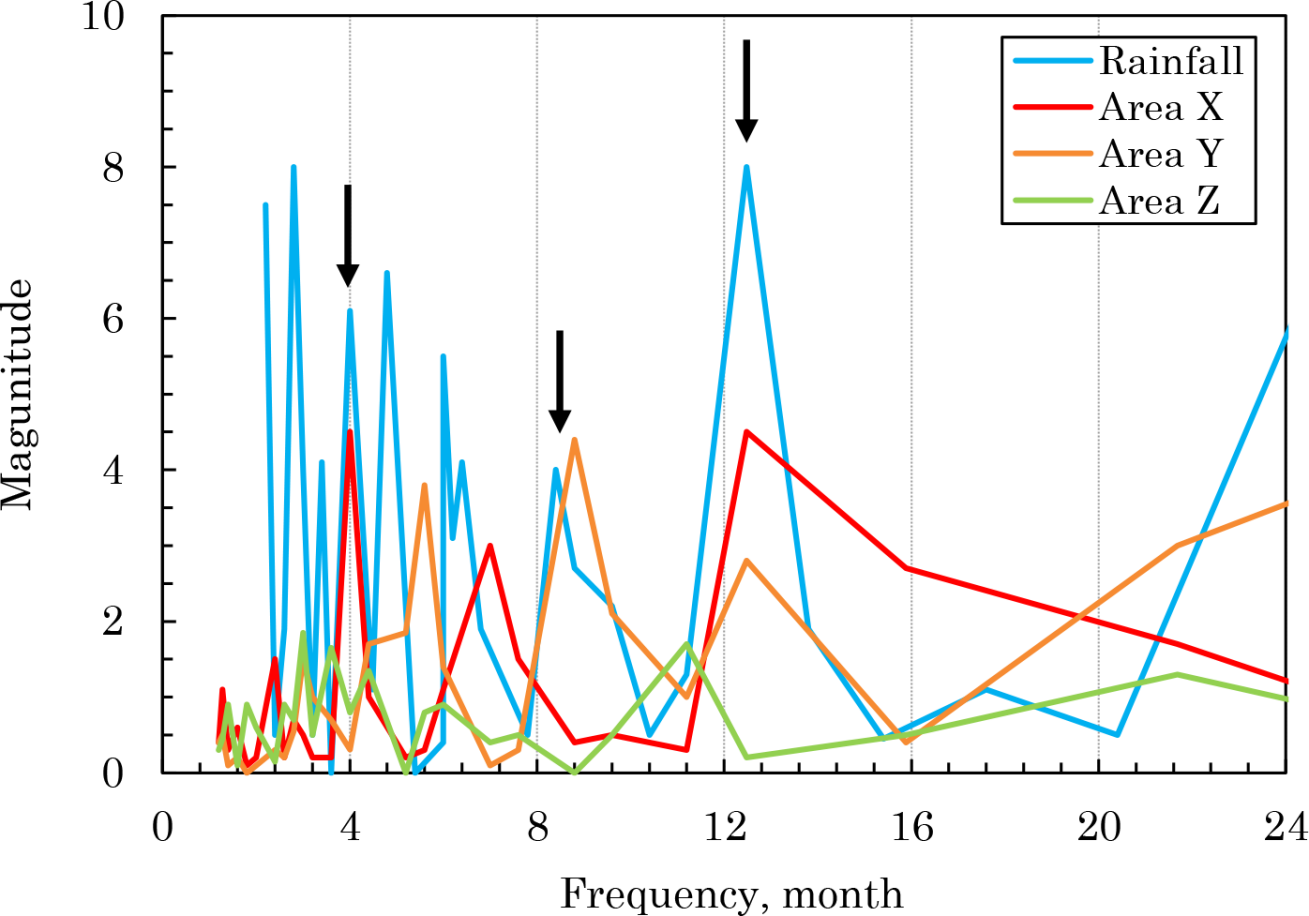
Comparison of the periodicity of the ^134+137^Cs radioactivity in the incineration ash and monthly precipitation. Frequency analysis was performed using a MATLAB software. Vertical arrows indicate matching periods.

As already stated, in the garden soil of Site ‘i’, the radiocesium activity in the soil fell abruptly in the immediate aftermath of the FDNPP accident. This phenomenon was also found in the school ground soils of Koto Ward in eastern Tokyo [55]. Even in the case of sludge incineration ash, as shown in Fig 6, with the monthly rainfall in the Tokyo metropolitan area, the rapid decline of the radiocesium activity in the immediate aftermath of the accident has been confirmed. However, the phenomenon cannot be easily explained based on run-off via rainfall from changes in rainfall levels in Tokyo [56]. This means that the rapid decline of the radiocesium activity in sludge incineration ash in the immediate aftermath of the accident can be explained by hypothesizing that highly mobile chemical species in the radiocesium that were deposited immediately after the accident flowed into and were discharged from wastewater treatment systems. We could not find these radiocesium species directly, but cesium-bearing microparticles alluded to by many researchers [57–62] are considered to be the highly mobile form of cesium that we estimated. As shown in Table 4, from 82–85% of the radiocesium discharged from wastewater treatment systems was discharged during the 10 months when the radioactivity fell rapidly in the immediate aftermath of the accident. Regarding the steady state period from October 2011 to the present, the periodicity was examined between rainfall and radiocesium activity in the sludge incineration ash using MATLAB. The results are presented in Fig 7, and show that in the urbanized X and Y districts, the rainfall and radiocesium activity conform in 4, 8, and 12 month cycles. This suggests that in these districts, radiocesium flowed into the wastewater treatment systems, carried by rain water. However, in district Z, which has a different urban structure, such periodicity was not found. This result suggests that the abrupt fall in the radiocesium activity observed in the immediate aftermath of the accident in garden soil in the center of Tokyo and in sludge incineration ash was not dependent on rainfall.

**Table 4:** Proportion of the migratory radiocesium discharged soon after the accident.

Area	Amount of Migratary ^134+137^Cs Bq·kg^-1^	^134+137^Cs in the Sharp Decline Period		
		Initial Amount Bq·kg^-1^	Discharged Amount Bq·kg^-1^	Discharged %
X	50000	30000	42000	84
Y	20000	13400	17000	85
Z	6000	3770	4900	82

Amount of migratory ^134+137^Cs was estimated from Fig 6 on March 16, 2011. Period of Sharp decline for ^134+137^Cs from immedeiately after the deposition to the December 2011. Initial amount was the measured radioactivity of the ash at May 2011. "Discharged %" indicates that the percentage of ^134+137^Cs discharged in the initial ten months from the total amount of ^134+137^Cs discharged by the wastewater treatment system from October 2011 to December 2016.

The adsorption behavior of cesium to suspended particles has been mentioned already [63]. Recently, extremely fine particles containing high activity of radiocesium discharged by the FDNPP accident have been detected in different regions [57–62]. It is assumed that radiocesium on such extremely fine particles does not exist as an ionic species, and hence it is unlikely that it can be maintained adsorbed in clay layers. If it is assumed that extremely fine particles containing radiocesium were deposited on the ground surface in the immediate aftermath of the accident, it is highly likely that it was more easily transported by falling rain or blowing wind than occur as ionic cesium that is easily held in soil. Furthermore, it is considered that radiocesium contained in such extremely fine particles is insoluble in water, so it is presumed that when radiocesium contained in these particles deposited on concrete or asphalt, it is selectively washed-out by rainfall. Quantitative data on the coverage of buildings, concrete and asphalt to the ground in areas X, Y, and Z could not be obtained. However, the period of decrease in radiocesium in the sludge incineration ash in areas X and Y is consistent with the fluctuating cycle in rainfall amounts. It can be thought that radiocesium deposited in these areas located in the central area of Tokyo is effectively decontaminated by rainfall. On the other hand, the radiocesium activity in the garden soil of Site ‘i’ located in area X, has not fluctuated significantly due to rainfall after the accident. Also, in the case of area Z, periodicity was not observed between rainfall and its radiocesium out-flow. This result indicates that not only the physical and chemical states of radionuclides but also the urban structure may have a significant influence on the behavior of radionuclides when urban areas, such as the Tokyo metropolitan area, receive radioactive contamination.

## Conclusions

Changes during the past five years in the distribution of environmental radioactive contamination in the Tokyo metropolitan area from the FDNPP accident were discussed. In the immediate aftermath of the accident, ^131^I, ^134^Cs, and ^137^Cs were detected in the soil of the Tokyo metropolitan area. High activities and inventories of the radionuclides were found in eastern Tokyo and northern Chiba. Inventories of decay corrected ^131^I and ^134+137^Cs on March 16, 2011 were 88–1010 kBq·m^-2^ and 11–93 kBq·m^-2^, respectively, which are about 40% and 20%, respectively, of those in Fukushima City. The contamination was even higher in the adjoining northern part of Chiba located east of Tokyo.

A rapid decline in the radiocesium activity in the immediate aftermath of the accident was observed in surface soil, suggesting the possibility that the radiocesium deposited on the ground surface occurred as highly mobile chemical species. By the end of 2016, approximately 85% of the spilled radiocesium in the wastewater treatment system flowed out during the 10 months after the accident. The run-off rate was estimated between 0.12–0.15%·day^-1^, signifying that the run-off half-life of radiocesium by wastewater treatment systems ranges from 460 to 580 days. From the radiocesium activity in the soil and the emissions as sludge incineration ash, it was estimated that 78–90% of the radiocesium deposited in the Tokyo metropolitan area still remains in the surface soil.

The radiocesium activity in the sludge incineration ash was higher in the urbanized eastern part of Tokyo, and there was correlation with changes in rainfall in that area. This suggests that radiocesium deposited on concrete and asphalt is more likely to be discharged than that on soil. The results reveal that the Tokyo metropolitan area even now continues to be affected by radioactive contamination caused by the FDNPP accident. We hope this study will contribute to the improved understanding of the radioactive contamination issue in Japan.

## Supporting information

S1 Fig Photos of site ‘i’. (PPTX)

S1 Table Analytical results of the activity of radionuclides in the soil samples collected in the Tokyo metropolitan area and the Kanto district area. Decay corrected activity was corrected on the March 16, 2011. Inventory was caluculated assuming 1,300 kg·m^-3^ for the soil density. At Site 'i', measurements were continued from April 10, 2011 until August 16, 2016. The results are shown in Fig 3. nd: Not Detected (^131^I; < 2 Bq·kg^-1^, ^134^Cs and ^137^Cs; < 0.6 Bq·kg^-1^).
(XLSX)

S2 Table Analytical results of the activity of radionuclides in the core samples collected in the site 'i'. Decay corrected activity was corrected on the March 16, 2011. Inventory was caluculated assuming 1,300 kg·m^-3^ for the soil density. nd: Not Detected (^131^I; < 2 Bq·kg^-1^, ^134^Cs and ^137^Cs; < 0.6 Bq·kg^-1^).
(XLSX)

S3 Table Temporal changes of analytical results of the activity of radionuclides in the incineration sludge ash in each wastewater treatment plant. nd: Not Detected. The data was refered to website of Tokyo Metropolitan Government. (http://www.gesui.metro.tokyo.jp/english/oshi/).
(XLSX)

S4 Table Annual amount of the incineration sludge ash collected from each wastewater treatment plant. The data was refered to website of Tokyo Metropolitan Government. (http://www.gesui.metro.tokyo.jp/business/pdf/2016tokyo.pdf (in Japanese)).
(XLSX)

S5 Table Monthly rainfall precipitation in the Tokyo mentropolitan area. The data was refered to website of Japan Meteorological Agency. (http://www.data.jma.go.jp/obd/stats/etrn/index.php?sess=6ef525a9cdef28cea634ce58ca736e68(in Japanese)).
(XLSX)
