# Supplementary figures and images for: Radioactive contamination in the Tokyo metropolitan area in the early stage of the Fukushima Daiichi Nuclear Power Plant (FDNPP) accident and its fluctuation over five years
Source: PLoS One. 2017 Nov 14;12(11):e0187687. doi: 10.1371/journal.pone.0187687 (PMC5685630; doi:10.1371/journal.pone.0187687)

## Slide 1
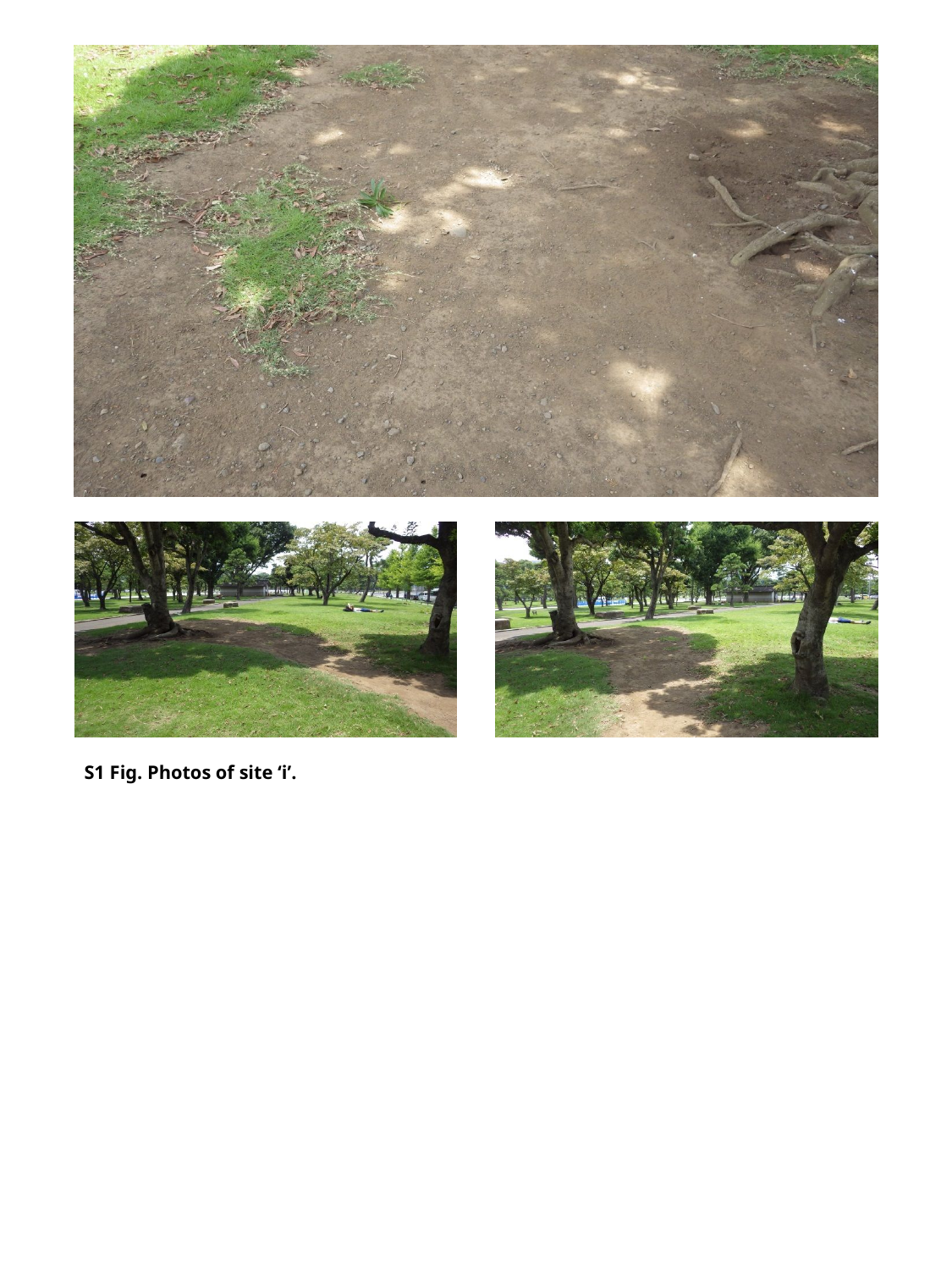

S1 Fig. Photos of site ‘i’.

Supplement: S1 Fig — (PPTX) [file pone.0187687.s001.pptx]
